# Effects of phosphorus-modified biochar as a soil amendment on the growth and quality of *Pseudostellaria heterophylla*

**DOI:** 10.1038/s41598-022-11170-3

**Published:** 2022-05-04

**Authors:** Charles Wang Wai Ng, Yu Chen Wang, Jun Jun Ni, Pui San So

**Affiliations:** Department of Civil and Environmental Engineering, The Hong Kong University of Science and Technology, Clear Water Bay, Hong Kong SAR China

**Keywords:** Plant sciences, Environmental sciences

## Abstract

Phosphorus (P) deficiency in agricultural soil is a worldwide concern. P modification of biochar, a common soil conditioner produced by pyrolysis of wastes and residues, can increase P availability and improve soil quality. This study aims to investigate the effects of P-modified biochar as a soil amendment on the growth and quality of a medicinal plant (*Pseudostellaria heterophylla*). *P. heterophylla* were grown for 4 months in lateritic soil amended with P-modified and unmodified biochar (peanut shell) at dosages of 0, 3% and 5% (by mass). Compared with unmodified biochar, P-modified biochar reduced available heavy metal Cd in soil by up to 73.0% and osmotic suction in the root zone by up to 49.3%. P-modified biochar application at 3% and 5% promoted the tuber yield of *P. heterophylla* significantly by 68.6% and 136.0% respectively. This was different from that in unmodified biochar treatment, where tuber yield was stimulated at 3% dosage but inhibited at 5% dosage. The concentrations of active ingredients (i.e., polysaccharides, saponins) in tuber were increased by 2.9–78.8% under P-modified biochar amendment compared with control, indicating the better tuber quality. This study recommended the application of 5% P-modified biochar for promoting the yield and quality of *P. heterophylla*.

## Introduction

Phosphorous (P) is an essential element for plant growth and productivity, due to its vital role in many basic metabolisms, including photosynthesis and respiration. The fixed P in soils is in the form of aluminum/iron or calcium/magnesium phosphates, which are generally not available for plant uptake^1^. Available P is scarce in 30–40% of the arable soils around the world^2^. Direct application of P fertilizers may lead to low P use efficiency due to the formation of precipitation and adsorption to soil particles^2^. Moreover, high P fertilizer usage could cause a higher risk of P loss through soil leaching, runoff and erosion, possibly leading to environmental issues like water eutrophication^3^. Therefore, how to reduce P loss and enhance soil P use efficiency deserves to be studied.

It is a potential approach to use some organic soil amendments like biochar to improve the soil P retention and therefore reduce P loss. Biochar is a carbon-rich material produced from the pyrolysis of biomass wastes at a high temperature with limited supply of oxygen. It has received wide attention due to its cost-effectiveness and environmentally-friendly nature. Biochar can improve soil fertility due to high levels of possessed nutrients^4^. Meanwhile, biochar could enhance water holding capacity and immobilize the potentially toxic metals through large specific surface area, increased micropores and oxygen-containing functional groups^3^. Therefore, plant growth and crop productivity can be enhanced. The feedstocks of biochar were mainly the residues and wastes in agriculture or industry^5^. For example, large peanut yield in China resulted in high production of peanut shell biochar for reutilization of resources^6^. However, a limitation in peanut shell biochar application is that the total P content is generally very low (< 1%)^2^. This may lead to unbalanced nutrient content, inhibiting seed germination and plant growth^7^. Therefore, it is necessary to improve P availability in biochar.

Recently, the modification of biochar with P has been found to improve P utilization efficiency and adsorption performance efficiently^3^. Based on the previous studies, P-modified biochar was produced via two main types of method. One type was to impregnate the feedstock into the solution of H_3_PO_4_ or K_3_PO_4_ and then pyrolyze it at a given temperature and duration^3,8^. Another way was to mix the pristine biochar with the solution of Ca(H_2_PO_4_)_2_∙H_2_O or KH_2_PO_4_ to lade it with P^9–11^. Subsequently, physicochemical characteristics and microstructures of the P-modified biochar were measured to evaluate the modification. More functional groups such as P–O, P–C groups were produced on biochar surface after modification, indicating biochar loaded by P^3^. Increased porosity in P-modified biochar contributed to a higher capacity to possess P when it was oxidated and immersed in phosphate solution. Consequently, when mixing P-modified biochar with soil, available P in biochar would release, causing a higher P concentration in soil for a long term^12^. Zhang et al.^3^ showed that P-modified biochar had a higher efficiency to immobilize Cu and Cd in soil. It was attributed to the increased adsorption ability with higher cation exchange capacity (CEC) and specific surface area after modification. Lyu et al.^13^ and Tan et al.^11^ pointed out the reduced leaching rate of uranium (U) under remediation by P-modified biochar. However, the previous studies mainly focus on the influence of P-modified biochar as a soil conditioner on the remediation of contaminated soil by changing physical and chemical properties^3,11,13^. The effects of P-modified biochar on the growth and quality of medicinal plants have not been studied.

Medicinal plants have abundant ingredients (e.g., terpenoids and polysaccharides), which can boost the development of medical therapy, commercial trade, human cultures and green ecology. *Pseudostellaria heterophylla* (*P. heterophylla*), belonging to the family of *Caryophyllaceae*, is one of the most widely used traditional Chinese medicinal plants. Its medicinal organ (i.e., root tuber) possesses various ingredients, including saponins, polysaccharides and amino acids. Those ingredients have multiple medicinal functions such as strengthening the spleen, replenishing Qi, moistening the lungs^14,15^. *P. heterophylla* is also selected to be one of the representative Chinese medicines for the prevention and treatment of Coronavirus Disease 2019 (COVID-19)^16^. However, the biomass and quality of its medicinal organ (root tuber) declines seriously due to soil sickness after replanting^17^. Besides, *P. heterophylla* is commonly planted in subtropical areas, such as Guizhou province in China, where cultivated soils are highly contaminated with heavy metals (e.g., Cd). Those heavy metals come from natural geological processes and anthropogenic activities, threatening the quality of medicinal plants and public health^18^. It is essential to promote the growth and quality of *P. heterophylla* by improving soil health and quality through soil remediation.

Therefore, the aim of this study is to investigate the effects of P-modified biochar treatment on the growth and quality of one popular medicinal plant *P. heterophylla*. Leaf area, shoot height, root properties and biomass of medicinal organ (root tuber) were measured to evaluate the growth of *P. heterophylla*. The concentrations of active ingredients (i.e., polysaccharides and saponins) in medicinal organs were also determined. This is the first study to reveal the yield and quality of medicinal plant (especially its medicinal organ) under P-modified biochar treatment with different dosages. The results have practical impacts on the instruction of large-scale planting.

## Results

### Soil physicochemical properties

**Figure 1:**
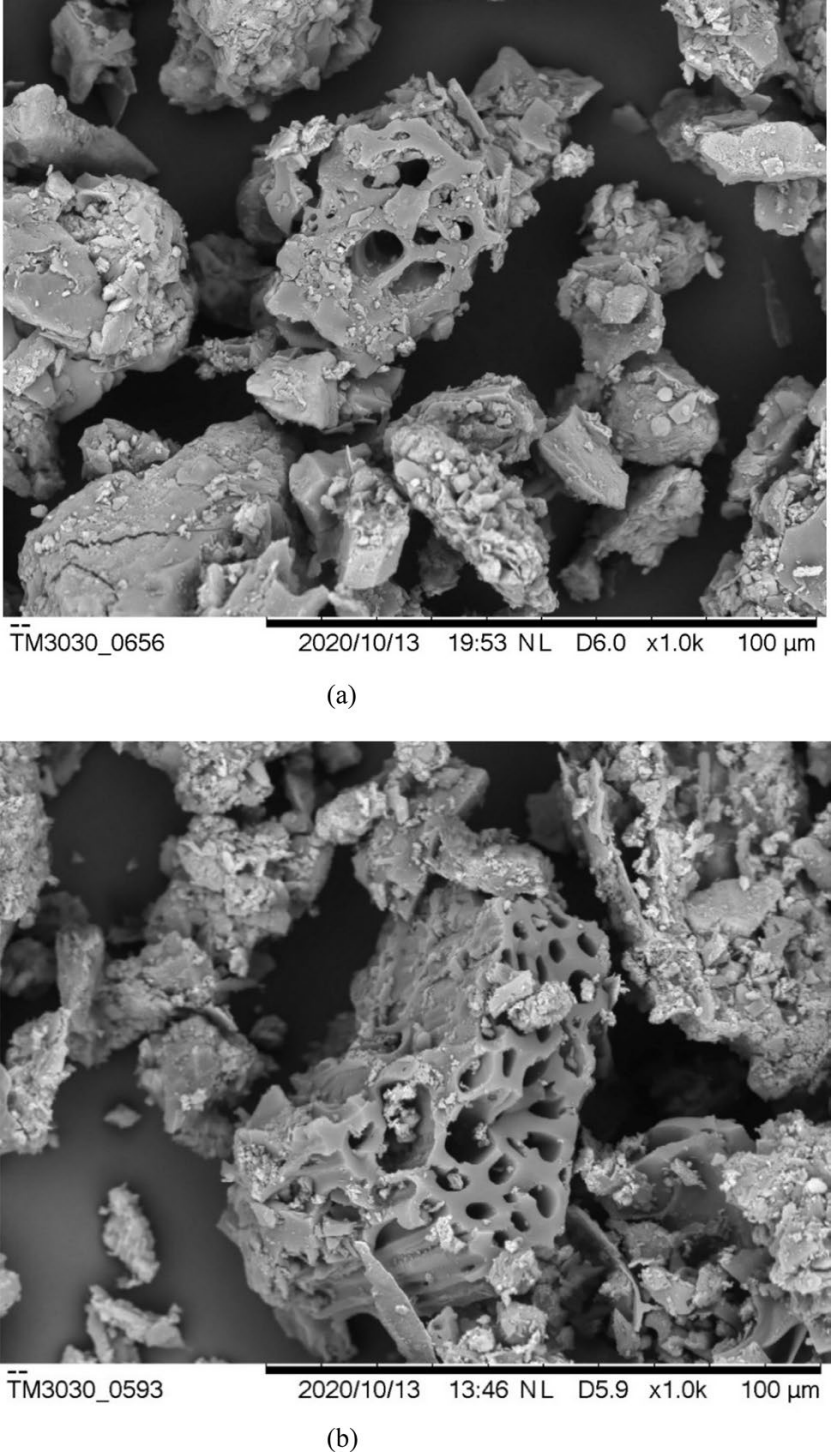
Scanning electron microscope (SEM) for (**a**) unmodified; (**b**) P-modified biochar.

Figure 1 shows the scanning electron microscope (SEM) results of unmodified and P-modified biochar. Their porous structure could be observed clearly. The number of pores was increased significantly after biochar modification, indicating the improved micropore system and larger specific surface area. Apart from micropore structure, the chemical compositions and functional groups were also altered under the biochar modification. The results of X-ray photoelectron spectroscopy (XPS) in Table S2 and its deconvolutions of P 2*p* etc. in Fig. S1 analyzed the chemical compositions of unmodified and P-modified biochar. The concentrations of some nutrient elements were increased in the biochar after P-modification from < 1 to 4.13% (mass concentration) for P and by 60.1% for K. Meanwhile, compared with unmodified biochar, the mass of some other elements such as Na, Al, Cl was reduced in P-modified biochar by 31.5–65.3%. According to Fig. S1, the peak energy of N 1*s* at 407.2–407.5 eV was lower for P-modified biochar. This illustrated that the content of NO_3_^−^ was decreased^19,20^. Meanwhile, by partially replacing NO_3_^−^, PO_4_^3−^ with a binding energy of 133 eV in P 2*p*^3,21^ was formed on biochar surface during modification. This was consistent with the peak at 531.0 eV in O 1*s* spectrum for P-modified biochar, which corresponded to the oxygen atoms of PO_4_^3−^ group^22^. Additionally, Fig. S2 shows that the peaks (e.g., 3420, 1030–1090, 500–600 cm^−1^) were strengthened for the P-modified biochar, compared with the spectrum for unmodified biochar. This indicates more hydroxy and PO_4_^3−^ were produced during the biochar modification^3,23^. This result was in agreement with Zhang et al.^3^ that P-modified biochar possessed more oxygen-containing functional groups such as hydroxy, P–O groups. The increased oxygen-containing functional groups in modified biochar was attributed to the oxidization of carbonized biochar surface under H_2_O_2_ treatment^24^. P impregnation by K_3_PO_4_ solution led to the PO_4_^3–^ laden biochar after modification. Increased hydroxy in modified biochar surface could enhance soil water retention capacity and immobilize soil contamination^3^. PO_4_^3−^ could improve soil P supply. Therefore, compared with unmodified biochar, P-modified biochar had more advantages in promoting plant growth.

Soil pH and nutrient elements (e.g., K, P, N) with significant changes after biochar amendment were shown in Table 1. Unmodified biochar at 3% and 5% could increase soil pH from 4.45 to 4.79 and 4.93 respectively. P-modified biochar increased pH from 4.45 to 5.11–5.33, which was above the minimum limit for the growth of *P. heterophylla*^25,26^. Soil available K increased significantly with the increasing dosage of biochar from 0 to 5% (*p* < 0.05), regardless of the unmodified and modified biochar. When the application dosage of biochar was 3–5%, available K in soil increased by 118.3–142.0% under P-modified biochar treatment, compared with that under unmodified biochar. After incubation of biochar amended soil for half a month, the concentration of soil available P was relatively low in control (0.87 mg/kg) and unmodified biochar treated groups (0.69–1.15 mg/kg). All of them were at an extreme scarce level based on Specification of Land Quality Geochemical Evaluation (DZ/T 0295—2016)^27^. However, when the soil was treated with P-modified biochar, the soil available P increased to 8.02 (scarce level) and 23.91 mg/kg (abundant level) at 3% and 5% biochar dosages, respectively.

**Table 1:** Initial levels of soil pH and plant available nutrients in biochar amended soil.

Treatment	pH	K (mg/kg)	P (mg/kg)	N (mg/kg)	S (mg/kg)	Cl (mg/kg)
CK	4.45	90.07 ± 7.86a	0.87 ± 0.08a	33.84 ± 1.87a	1.16 ± 0.04a	36.16 ± 3.11a
B3	4.79	268.28 ± 4.39b	0.69 ± 0.12a	72.59 ± 2.17b	5.02 ± 0.59b	464.56 ± 10.49d
PB3	5.11	585.65 ± 10.12d	8.02 ± 1.12b	68.11 ± 2.73b	15.57 ± 0.82d	346.38 ± 4.31b
B5	4.93	400.40 ± 6.24c	1.15 ± 0.27a	102.06 ± 4.24c	12.84 ± 0.43c	793.21 ± 7.86e
PB5	5.33	968.93 ± 12.86e	23.91 ± 2.56c	93.53 ± 3.45c	46.12 ± 1.72e	397.45 ± 4.75c

The concentrations in this table are the values for plant available nutrients, including K (as K^+^, etc.), P (as H_2_PO_4_^−^, HPO_4_^2−^, PO_4_^3−^, etc.), N (as NH_4_^+^, NO_3_^−^, etc.), S (as SO_4_^2−^, etc.), Cl (as Cl^−^, etc.). CK, the group without biochar application; B3 and B5, the groups subjected to unmodified biochar application at 3% and 5% (by mass); PB3 and PB5, the groups subjected to P-modified biochar application at 3% and 5% (by mass). Data are presented as mean value ± standard deviation (n = 3). Different letters above the bars indicate significant differences (*P* < 0.05) between the groups under different treatments.

In addition, the concentration of N (mainly NH_4_^+^ and NO_3_^−^), S (SO_4_^2−^) and Cl (Cl^−^) increased significantly under biochar application, due to direct release from biochar to soil water solution^28,29^. Compared with the soils under unmodified biochar amendment, the concentrations of N and Cl in soil amended with P-modified biochar at the same dosage were reduced by 6.2–8.4% and 25.4–49.9% respectively. In contrast, the concentration of plant available S (i.e., SO_4_^2−^) under modified biochar amendment was increased. Hence, compared with unmodified biochar, P-modified biochar with higher nutrient (i.e., K, P, S) level and lower Cl^−^ concentration was more suitable for medicinal plant growth.

**Figure 2:**
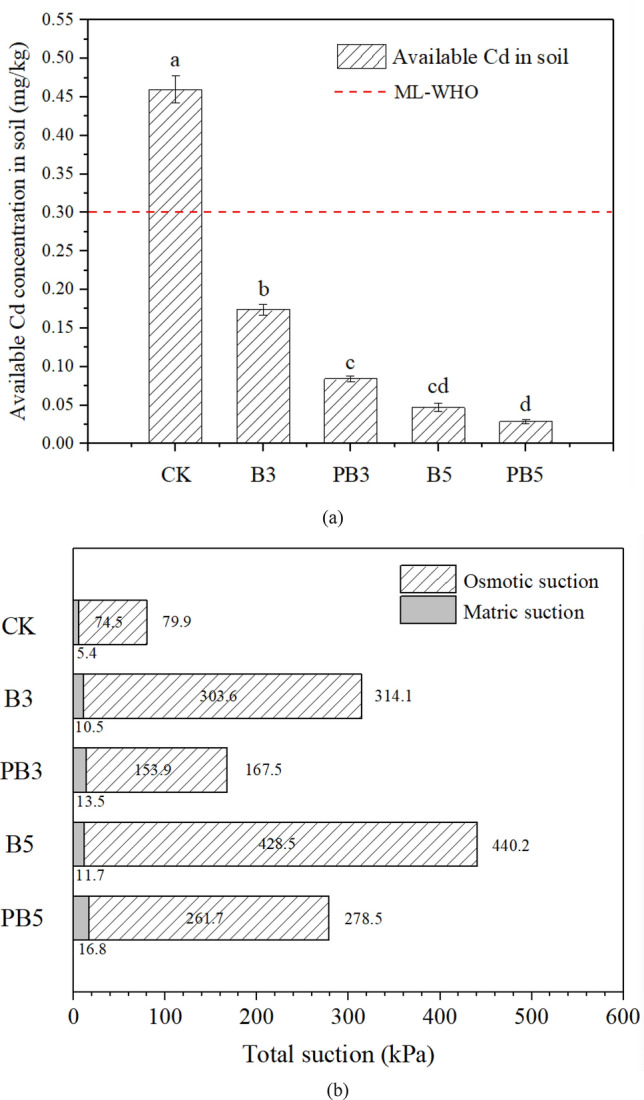
(**a**) Initial concentrations of plant available toxic metal Cd in soil and (**b**) mean total suction in soil (root zone) during plant growth period subjected to different amendments. Data are presented as mean value ± standard deviation (n = 3). Different letters above the bars indicate significant differences (*P* < 0.05) between the groups under different treatments.

Figure 2a shows the concentrations of available Cd in soil under different treatments. In the control group without biochar amendment, plant available Cd was 0.460 mg/kg, exceeding the permissible limits (0.3 mg/kg) proposed by the World Health Organization^30^. Both unmodified and modified biochar at 3–5% dosage could reduce soil available Cd significantly below the limit (0.3 mg/kg) (*p* < 0.05). Compared with unmodified biochar treatment, available Cd decreased from 0.174 to 0.084 mg/kg at 3% P-modified biochar dosage while that decreased from 0.047 to 0.029 mg/kg at 5% dosage.

Total suction in the root zone is also an important indicator of plant growth. Figure 2b shows the results of total soil suction in the root zone, which is the sum of matric suction and osmotic suction. Total soil suction in the root zone increased from 79.9 kPa for control soil to 314.1 kPa for 3% unmodified biochar treated soil. Total suction became higher with the increasing dosage of biochar. In the case of the soil treated with 5% unmodified biochar, the total suction in the root zone rose to 440.2 kPa. The total soil suction was reduced by 36.7–46.7% under 3% and 5% P-modified biochar treatments, respectively, compared with unmodified biochar treatment. In terms of matric suction, the average matric suction in the root zone during the plant growth period showed a slight increase from 5.4 to 10.5–11.7 kPa under unmodified biochar amendment. It increased to 13.5–16.8 kPa when the soil was treated by P-modified biochar. Slightly higher matric suction under biochar amendment was possibly due to improved plant growth and higher root water uptake ability^31^. Compared with the magnitude of matric suction, osmotic suction had a dominant role in affecting total suction in this figure. Therefore, it showed the similar effects of different soil treatments on total suction in the root zone. Unmodified biochar amendment increased osmotic suction by 307.5–475.2% at 3–5% application. Compared with unmodified biochar treatment, P-modified biochar reduced osmotic suction by 38.9–49.3%.

### Plant growth under different treatments

**Figure 3:**
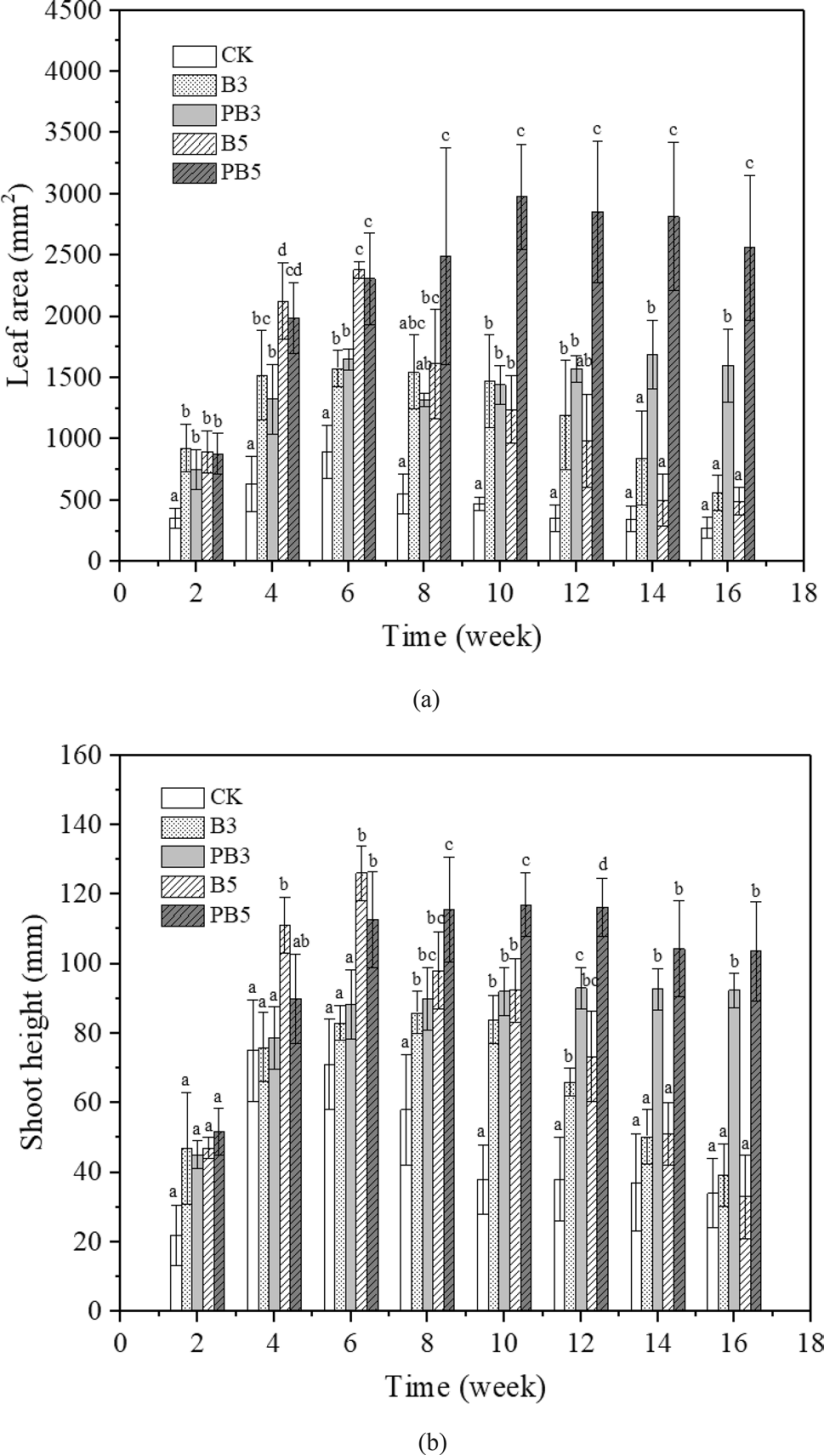
Variations of (**a**) leaf area and (**b**) shoot height during plant growth period (4 months). Data are presented as mean value ± standard deviation (n = 3). Different letters above the bars indicate significant differences (*P* < 0.05) between the groups under different treatments.

Figure 3 shows the leaf area and shoot height of *P. heterophylla* during the 4-month growth period (16 weeks). In Fig. 3a, leaf area increased by 70.6–165.7% until the 6–8th week and then decreased by 64.3–79.4% for control and unmodified biochar (3% and 5%) groups. However, for P-modified biochar groups (3% and 5%), it shows only slight decrease of leaf area after the 10th week. In the first 6 weeks, compared with control, 75.9–234.3% larger leaf area was observed in the groups with biochar application, regardless of biochar modification. There was no significant difference between unmodified and P-modified biochar treated groups at the same biochar dosage. After the 6th week, as the decrease of leaf area for control and unmodified biochar groups, the advantages of P-modified biochar to increase leaf area were highlighted. After the 10th week, significant enlargement of leaf area was observed in the P-modified biochar group compared with unmodified biochar groups (*p* < 0.05). Similar trends were also observed for shoot height in Fig. 3b. P-modified biochar amendment at the dosage of 5% was the most beneficial to the plant growth characteristics (leaf area and shoot height). One difference was that at the first 6 weeks, the shoot height of *P. heterophylla* under unmodified biochar treatment was higher (< 18.9%) than those under P-modified biochar at the same dosage.

Mean root length (RL) and root length density (RLD) are two morphological parameters to reflect the growth of underground parts, which are shown in Table 2. Root length among all treatments had no significant difference (*p* > 0.05). Root length density increased under all biochar amendments except 5% unmodified biochar treatment, which showed a reduction of 13.1% compared with control. At 3% biochar dosage, unmodified and P-modified biochar enhanced root length density by 3.3% and 50.5% respectively. Compared with control, the most significant increase of root length density by 61.1% was in the group under 5% P-modified biochar treatment (*p* < 0.05).

**Table 2:** Changes in morphological parameters of *P. heterophylla* root in response to biochar amendment.

Treatment	RL (mm)	RLD (m/m^3^)
CK	58.4 ± 4.8a	1986.6 ± 118.1a
B3	56.3 ± 1.7a	2051.5 ± 168.0a
PB3	61.8 ± 7.4a	2989.6 ± 641.1ab
B5	55.9 ± 6.3a	1726.5 ± 400.8a
PB5	65.9 ± 7.2a	3201.3 ± 469.8b

*RL* root length, *RLD* root length density.

Data are presented as mean value ± standard deviation (n = 3). Different letters above the bars indicate significant differences (*P* < 0.05) between the groups under different treatments.

### Yield and quality of root tuber

**Figure 4:**
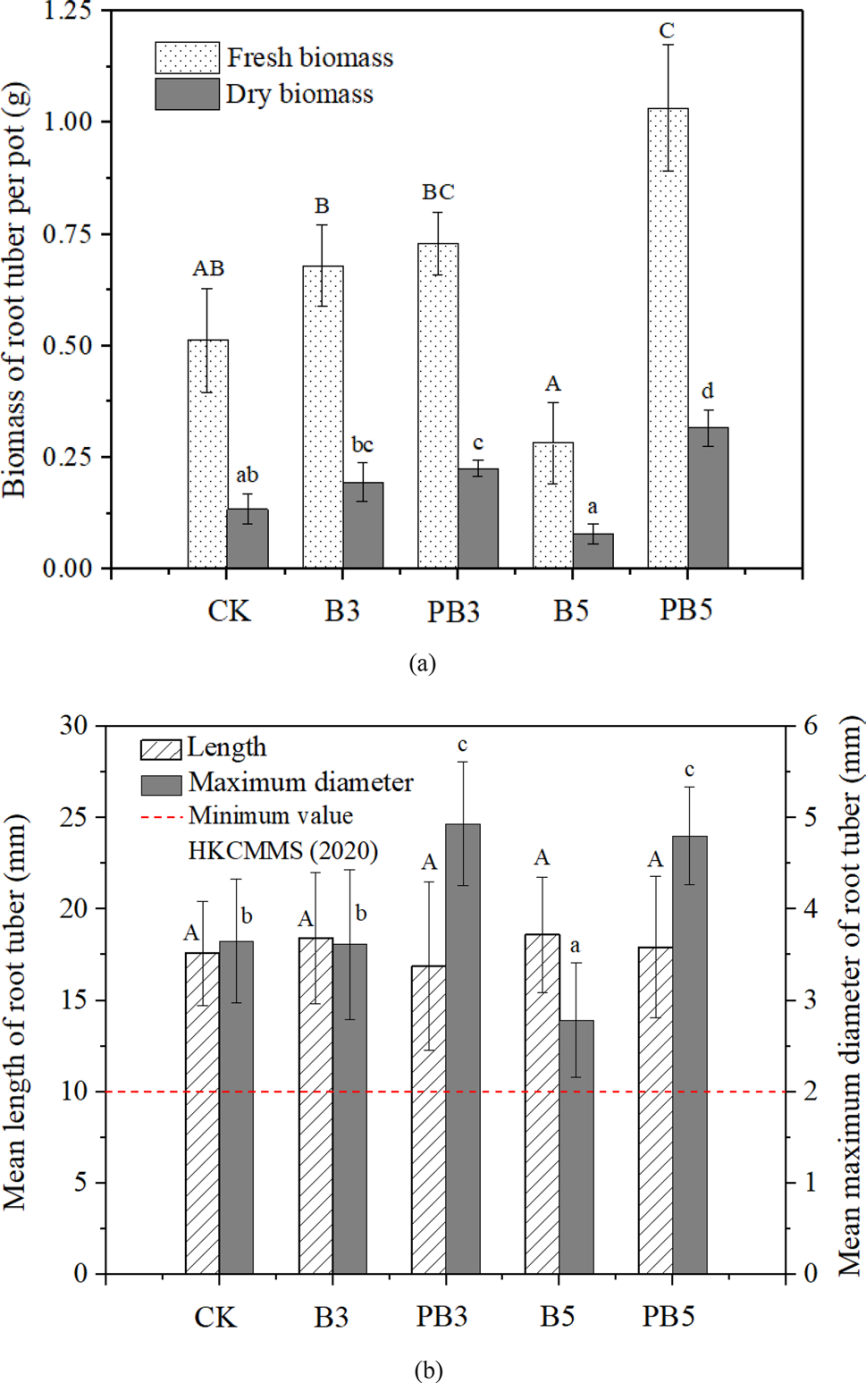
Variations of (**a**) biomass and (**b**) morphology (mean length and mean maximum diameter) of root tuber. Data are presented as mean value ± standard deviation (n = 3). Different letters above the bars indicate significant differences (*P* < 0.05) between the groups under different treatments.

Figure 4a shows the fresh and dry biomass of root tuber under control, unmodified and P-modified biochar treatments. Application of 3% unmodified biochar increased the tuber biomass by 45.4% (from 0.134 for control to 0.195 g/pot) (*p* > 0.05). When the application dosage increased to 5%, the dry biomass of root tuber decreased to 0.079 g/pot. As for P-modified biochar amendment, it was significantly increased by 68.6% and 136.0% at 3% and 5% dosages, respectively, compared with control *(p* < 0.05). At the 3% and 5% dosages of biochar application, P-modified biochar enhanced the yield (dry biomass) by 16.0% (*p* > 0.05) and 301.0% (*p* < 0.05) respectively, compared with unmodified biochar. The similar trends were also observed in fresh biomass.

Figure 4b shows the morphology of *P. heterophylla* tuber including the mean length and mean maximum diameter under different soil treatments. The difference of tuber length under various treatments including control showed no statistical significance. The mean maximum diameter of root tuber declined from 3.65 mm for control to 2.79 mm for 5% dosage of unmodified biochar. Unlike unmodified biochar, P-modified biochar increased the mean tuber diameter by 35.2% and 31.5% to 4.93 mm and 4.80 mm, at dosages of 3% and 5% respectively (*p* < 0.05).

**Figure 5:**
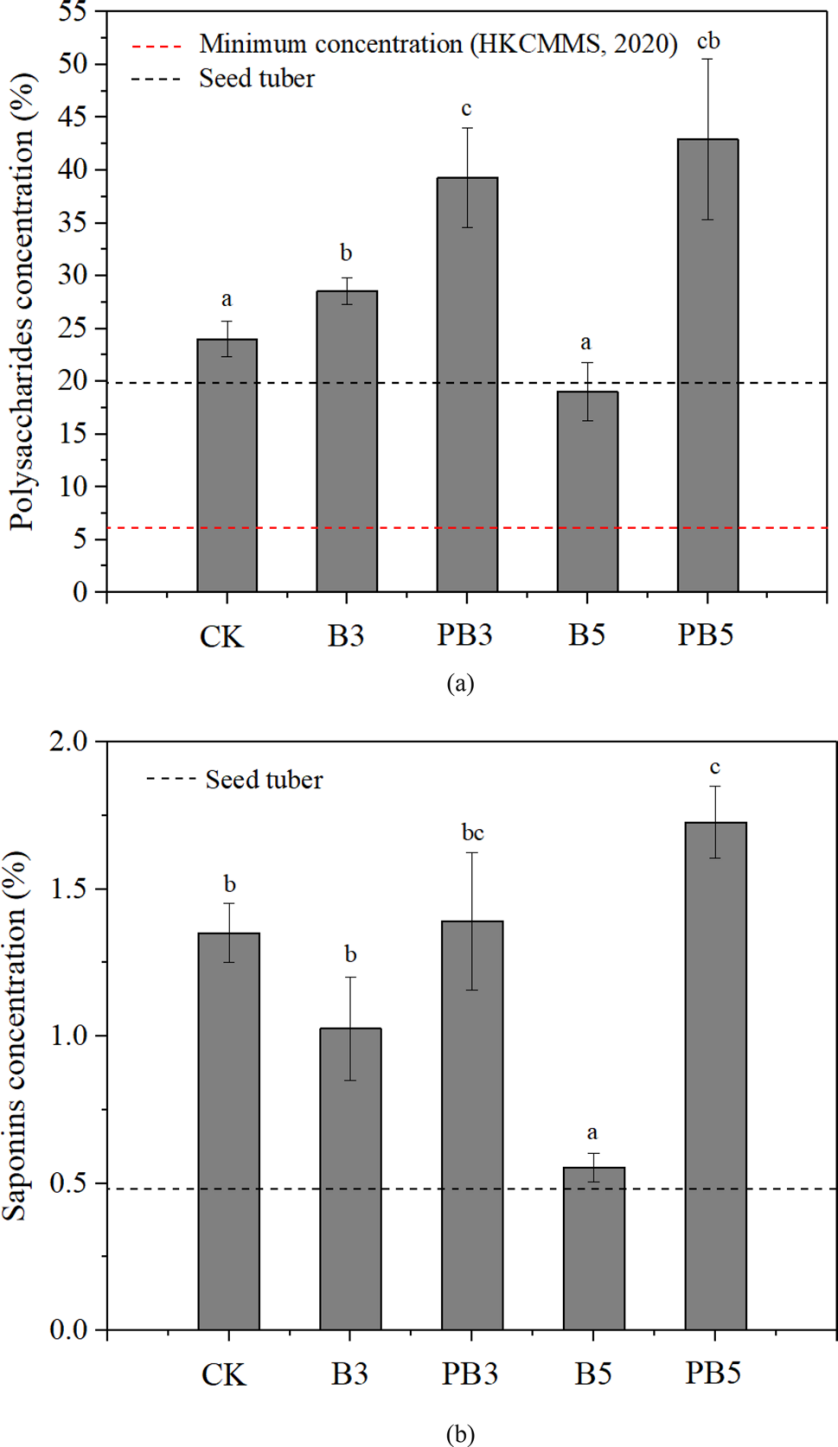
Active ingredients including (**a**) polysaccharides and (**b**) saponins in root tuber. Data are presented as mean value ± standard deviation (n = 3). Different letters above the bars indicate significant differences (*P* < 0.05) between the groups under different treatments.

The concentration of active ingredients in the medicinal organ is one of the key factors to evaluate the quality of medicinal plants. Figure 5 shows the active ingredients (polysaccharides and saponins) of the dried root tuber of *P. heterophylla*. In Fig. 5a, compared with control, application of 3% unmodified biochar improved the polysaccharides concentration in root tuber by 19.0% while 5% unmodified biochar application decreased it by 20.7%. For P-modified biochar amendment, the concentrations of polysaccharides were increased significantly by 63.7% and 78.8% at 3% and 5% dosages respectively, compared with control (*p* < 0.05). The concentration of polysaccharides in dried root tuber under all these treatments was above the minimum limit of 6% proposed in HKCMMS (2020)^32^. Compared with the seed tuber, polysaccharides concentration was increased by 20.6–115.7% under all biochar treatments except at 5% unmodified biochar, in which it was decreased by 4.4%. The concentration of saponins, another important active ingredient for *P. heterophylla*, was measured and the results are shown in Fig. 5b. Compared with control, the 3% and 5% dosages of unmodified biochar decreased the concentrations of saponins in dried root tubers by 24.1% and 59.0%, respectively. In contrast, saponins concentrations were increased by 2.9% and 27.8% at 3% and 5% P-modified biochar treatments, respectively. The application of 5% P-modified biochar could improve saponins concentration with statistical significancy (*p* < 0.05). The root tuber under all these treatments enhanced the concentrations of saponins by 15.5–260.0% compared with their seed tuber.

### Relationships between soil conditions and plant properties

**Figure 6:**
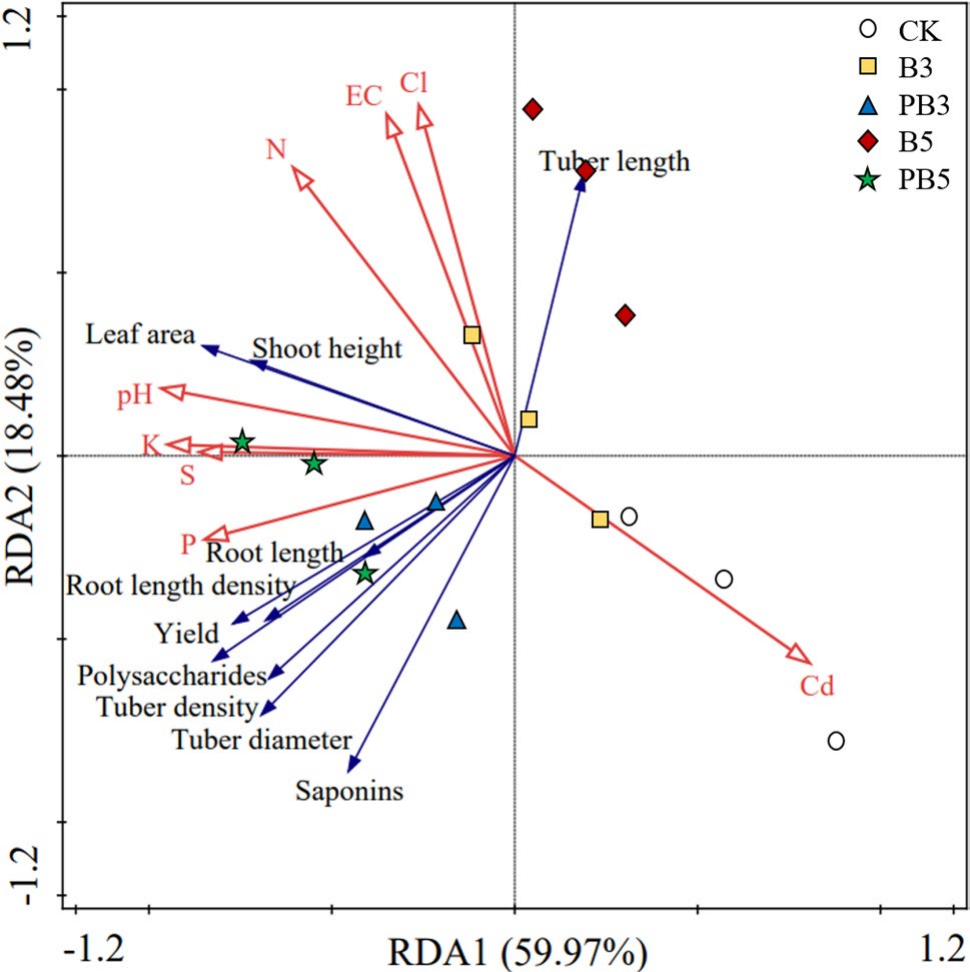
The relationships among the plant characteristics (blue arrow with black font) and soil physicochemical parameters (red arrow) measured in five groups under different treatment based on a redundancy analysis (RDA). The first two RDA components could explain 78.5% (59.97% + 18.48%) of the total variation.

Figure 6 shows the relationships between plant characteristics and soil physicochemical properties assayed by RDA. The first two axes of the RDA could explain 78.5% of the total variation. The first component (RDA1) largely differed for soil nutrient levels (e.g., P, K, S) and soil toxic metal (i.e., Cd), separating the P-modified biochar treatments (blue and green points) from control. The second component (RDA2) mainly explained the variability driven by soil EC and Cl concentration, separating unmodified biochar treatments (yellow and red points) from others. As shown by the vectors in RDA, soil pH and nutrient levels (K, P, S) were positively related to most plant characteristics except tuber length. It may be due to the elongation of the underground part for the acquisition of more nutrients like P by the plant under P deficiency^33^. Among the soil nutrient elements, P showed the closest relationship with properties of root and tuber while K, S, N had more significant effects on leaf and stem of *P. heterophylla* than other nutrients. Therefore, P, which was mainly supplied by P-modified biochar, was a promising nutrient to improve yield and active ingredients of *P. heterophylla* tuber with relatively high efficiency, followed by S and K. Cd concentration was negatively correlated with most plant characteristics especially leaf area and shoot height due to its metal toxicity. However, it showed no negative effects on the concentration of saponins in the tuber, due to potential stimulation of Cd on plant defense for saponins biosynthesis^26^. Soil EC and Cl showed negative correlations with properties of root and tuber but positive correlations with tuber length of *P. heterophylla*.

## Discussion

According to the results from XPS (in Table S2, Fig. S1), FTIR (in Fig. S2) and the measurements of available nutrients (in Table 1), the supply and availability of K and P were increased by P-modified biochar. This was mainly due to the application of K_3_PO_4_ solution for P source during biochar modification. More pores were created when biochar was oxidated by diluted H_2_O_2_ solution in modification procedure (SEM in Fig. 1), leading to the biochar with larger surface area and hence enhanced possession of K and P^12^. P was adsorbed onto the modified biochar surface by pore filling, electrostatic attraction, anion exchange, surface precipitation, etc^34^. After mixing modified biochar with soil, the possessed K and P by biochar during modification could be re-released into soil water directly as a nutrient source^35^. This process was governed by solute diffusion under concentration gradient from biochar phase into the aqueous phase^36,37^. The repeatable released P in soil water was transported to the root surface by mass flow or diffusion for plant uptake^38^. Unmodified biochar at 5% dosage could also slightly increase the P availability due to the soil neutralization by biochar in acid soil to reduce the constraint of soil exchangeable Al on soil available P^2^. However, it was partially counteracted by the potentially increased soil P sorption under unmodified biochar amendment. Therefore, the improvement of P availability by unmodified biochar was not statistically significant. Due to the relatively low concentration of available P (0.69–1.15 mg/kg) in control and unmodified biochar treated soil (Table 1), the diffusion of P to rhizosphere is limited. Therefore, the nutrients (i.e., P) absorbed by plant via root water uptake could not meet the plant nutrient demand, leading to reduced leaf area, shoot height, RL and RLD^39^. P-modified biochar (3–5%) enhanced available P concentration (8.02–23.91 mg/kg) in soil. Once the P concentration in the root zone was reduced due to plant uptake, it would be compensated from the non-rhizosphere region based on nutrient gradient^40^. After 4-month plant growth, the available P level in the rhizosphere of *P. heterophylla* under P-modified biochar treatment was 4.73–13.70 mg/kg at harvest. It was still obviously higher than that under the control and unmodified biochar treated groups (i.e., < 3 mg/kg). This indicated that P-modified biochar could desorb P into soil water as a supplement in a relatively long term (i.e., more than 4 months). This was consistent with the finding by Zhang et al.^12^ that P-modified biochar could increase the soil available P from < 20 mg/kg to approximate 39 mg/kg and remain this available P level for more than 45 days.

Apart from improved soil fertility (e.g., P), the reduced stress caused by toxic metal (Cd) and high soil total suction was another reason for plant growth promotion by P-modified biochar (Fig. 3), compared with unmodified one. In terms of Cd stress (Fig. 2a), firstly, the increase of pH value in soil amended with biochar especially P-modified one (Table 1) was one important reason for Cd immobilization. Based on the negative correlation between pH and Cd eluate from soil proposed by Monhemius et al.^41^, the application of modified biochar could lead to Cd immobilization with higher efficiency in soil. Secondly, more oxygen-containing functional groups (e.g., hydroxy, carboxyl) in P-modified biochar^3^ increased the negative charge of amended soil, leading to better adsorption ability to Cd. Relatively abundant P in modified biochar amended soil might form some precipitations like Cd(PO_3_)_2_, Cd(OH)PO_3_·H_2_O with Cd, resulting in a reduction of Cd availability^3^. Lastly, increased number of micropores and larger specific surface area of P-modified biochar (SEM in Fig. 1) resulted in more available Cd adsorbed on the surface or into the pores. This led to reduced Cd mobility in soil and enhanced soil quality. Meanwhile, the growth of plants in soil treated by P-modified biochar could be improved due to reduced Cd stress with less phytotoxicity and ecotoxicity. Available soil Cd could induce stomata closure, decrease the content of chlorophyll and inhibit the root elongation, leading to reduced plant growth^26^. Therefore, P-modified biochar reduced Cd stress and hence promoted the growth of both aboveground and underground part efficiently. Increase of soil pH was another reason for plant growth improvement by biochar treatment especially P-modified biochar. Soil acidity and toxicity of excessive H^+^ and Al^3+^ were alleviated, reducing negative effects on root elongation^42^.

Based on the previous study, biochar possessed abundant nutrients (K, N, P, Ca, S, Mg, etc.)^43^. Increased nutrient ions (e.g., available K^+^) released from biochar into soil water, leading to a higher osmotic suction under both unmodified and P-modified biochar amendments^44,45^. Compared with unmodified biochar, some ions (e.g., Cl^−^) were reduced significantly during washing and filtering processes in modification (Table 1). It could result in a lower concentration of anions in soil water. In addition, increased micropores in biochar after modification (SEM in Fig. 1) could improve the adsorption capability for ions (e.g., Na^+^)^46^, leading to osmotic suction reduction and therefore decreased total suction. As pointed out by Ng et al.^47^, the rate of plant water uptake was governed by Darcy’s law, which is defined as the product of hydraulic gradient and hydraulic conductivity. Hydraulic gradient is proportional to the difference of hydraulic heads (i.e., controlled by total suction) between the plant roots and soil. A high total suction in an unmodified biochar amended soil reduced the hydraulic gradient between soil and plant roots. Hydraulic conductivity of plant roots was also reduced in the soil with high total suction^48^. Root water uptake was sequentially inhibited^47^. Inhibited water uptake by roots in soil with high total suction also reduced plant growth (e.g., root elongation) and metabolism (e.g., photosynthesis)^49^. Therefore, compared with P-modified biochar or unmodified biochar at relatively low dosage (3%), 5% unmodified biochar with higher total suction in the root zone led to the decline in tuber yield. P-modified biochar decreased the total suction in the root zone and potentially enhanced water uptake, contributing to the promoted growth of *P. heterophylla*.

An excess of Cl concentration in unmodified biochar was another possible reason leading to a shorter plant growth period and less plant growth, compared with P-modified biochar. Excessive Cl in soil could result in inhibited plant uptake of some nutrient anions (e.g., NO_3_^-^, H_2_PO_4_^−^) by roots through antagonism, contributing to a limited improvement of plant growth^50^. It also induced the burn of leaf tip with limited photosynthesis^51^. As the plant grew, the reduced nutrient levels and the continued uptake of Cl caused by transpiration resulted in higher Cl accumulation in the plant with more severe toxicity^52^. Therefore, P-modified biochar with reduced Cl content showed improved plant growth characteristics.

Improved shoot growth with enlarged leaf area (Fig. 3) under P-modified biochar treatment enhanced the photosynthetic capacity, producing more carbohydrates. Apart from the organic compounds utilized by plant growth and metabolism, others as storage products were transferred and accumulated in the organs like root tuber^53^. Due to the increased storage of carbohydrates into tuber via promoted photosynthesis, the root tuber growth of *P. heterophylla* was improved in P-modified biochar treated groups^54^. As for unmodified biochar treated groups, high dosage (i.e., 5%) application reduced leaf area after the 6th week. Therefore, declined leaf growth at a relatively early period led to the lower storage of carbohydrates in root tuber and hence less yield of *P. heterophylla*. In addition, the up-regulated plant hormones for the formation of roots (including adventitious root) by biochar possibly contributed to increased tuber yield^55^.

The polysaccharides concentration in root tuber was positively correlated to plant growth and yield, based on Fig. 6. The previous study pointed out that the highest biomass accumulation and polysaccharide production were also simultaneously observed in the same condition for another medicinal plant *Callerya speciosa*^56^. Polysaccharides were polymeric carbohydrate macromolecules in the plant, which were derived from carbohydrate via carbon reactions of photosynthesis^57^. Promoted photosynthesis produced more carbohydrate as the feedstock for the synthesis of polysaccharides, possibly leading to higher polysaccharides concentration in the tuber. Therefore, inhibited leaf growth and water uptake under 5% unmodified biochar caused by high total suction in the root zone led to the reduced photosynthesis and hence polysaccharides concentration in tuber. Compared with unmodified biochar, P-modified biochar supplied higher levels of nutrients (e.g., P) and reduced the total suction in the root zone, resulting in the enhanced polysaccharides accumulation and therefore higher plant quality. The declined concentration of saponins in *P. heterophylla* tuber with increasing dosage of unmodified biochar is likely due to regulated gene expression under biochar amendment^55^. Viger et al.^55^ found out that biochar addition could induce the down-regulation of genes related to plant defense like biosynthesis of jasmonic acid and secondary metabolites. Therefore, reduced stimulation of saponins formation by inhibited jasmonic acid resulted in decreased saponins concentration in tuber.

However, P modified biochar improve the synthesis of active ingredients (polysaccharides and saponins) of *P. heterophylla* in tuber in two possible ways. Firstly, P (e.g., phosphate) supplied by P-modified biochar (shown in XPS, FTIR results, etc.) had a vital impact on the accumulation of active ingredients (polysaccharides and saponins) directly^58^. Yin et al.^59^ concluded that the accumulation of polysaccharides and saponins for the adventitious roots of *P. heterophylla* was enhanced as phosphate supply increased from 0 to 2.5 mM. Plant P uptake might play an important role in the formation of some enzymes or precursors related to saponins synthesis^60^. Relative abundant P supply and plant uptake under the P-modified biochar treatment stimulated the synthesis of active ingredients in tuber of *P. heterophylla* and therefore improved its quality. Indirectly, morphology of root tuber changed by biochar amendment could also affect the accumulation of polysaccharides and saponins. The plumper tubers with less length and larger diameter under P-modified biochar treatment tended to have higher concentration of active ingredients than the slender ones in control and unmodified biochar treated groups^26^.

## Conclusions

In conclusion, P-modified biochar enhanced plant available P in soil. Meanwhile, it decreased the stress of potentially toxic elements (i.e., Cd, Cl) and high soil osmotic suction, compared with unmodified biochar treatment. P-modified biochar showed higher efficiency in promotion of *P. heterophylla* yield by 16.0–301.0% in comparison to unmodified biochar. This indicated that P-modified biochar could minimise the negative effects caused by high dosages of unmodified biochar and supply more nutrients for plant uptake. Compared with control and unmodified biochar treated groups, both the active ingredients (i.e., polysaccharides and saponins) in tuber were enhanced by P-modified biochar significantly. Therefore, this study reveals that P-modified biochar is a promising soil amendment in herbal planting. An application of 5% P-modified biochar by mass is recommended to enhance the growth and quality of *P. heterophylla* with higher yield and active ingredients production. In the further study, it is necessary to carry out more analyses (XRD, etc.) of modified biochar to deepen the understanding of its altered properties and characterizations. In addition, a larger number of replicates for laboratory experiments and field test should be considered for further large-scale cultivation.

## Methods

### Tested soil properties

The tested lateritic soil was collected from Bijie, Guizhou province (27° 24′ N, 105° 20′ E), which is the top producer and exporter of Chinese medicine in China. After the field soil was air-dried, it was sieved through a 4.75 mm mesh for planting. The pH of the tested soil sample was measured by pH meter following ASTM D4972-01 (2007)^61^. Electric conductivity (EC) of soil samples was determined by conductivity meter^62^. Cation exchange capacity (CEC) was analysed following the method proposed by Gillman and Sumpter^63^. Total carbon and inorganic carbon in biochar were measured by a total organic carbon analyzer (Shimaszu, TOC-VCPH). The plant available nutrients (i.e., K, P, Mg, Ca, Cu, Zn) in soil samples were extracted by Mehlich 3 Extraction Method^64^. The detailed basic physicochemical properties of the tested soil are summarised in Supplementary Table S1 in Supplementary Information.

### Phosphorus modification of biochar and biochar amended soil

Biochar was supplied by Sanli New Energy Co., Ltd. in Shangqiu, Henan province and it was produced from peanut shell at 500 °C pyrolysis. After being sieved through a 2 mm mesh, the contents of biochar with the particle size of < 0.075 mm, 0.075–0.425 mm, > 0.425 mm were 7.7%, 48.1% and 44.2% respectively. The production of phosphorus-modified biochar (P-modified biochar) was based on Zhang et al.^12^. Firstly, 10 g/L potassium phosphate (K_3_PO_4_) solution was prepared, which was mixed with the diluted hydrogen peroxide (8% by mass). The mixed solution was added to the crushed and sieved (< 2 mm) biochar according to the mass ratio of 2:1 for about 24 h. Hydrogen peroxide was used in this procedure for oxidization to increase the biochar surface area and the P adsorption. After filtration, the modified biochar was dried in an oven at a temperature of 60 °C for 24 h. After cooling it to room temperature, the modification process was finished. The scanning electron microscope (SEM) results of unmodified and P-modified biochar were present in Fig. 1. It is observed that the increased number of pores in biochar after modification. X-ray photoelectron spectroscopy (XPS) was used to analyze the chemical compositions of unmodified and P-modified biochar and the results are presented in Supplementary Table S2. XPS deconvolutions of N1*s*, P2*p*, O1*s*, C1*s* were also performed and the results are presented in Supplementary Fig. S1. The surface functional groups of biochar before and after modification were determined by a Fourier transform infrared spectroscopy (FTIR). After baseline correction, the FTIR spectra are shown in Fig. S2.

Both the unmodified (B3, B5) and P-modified biochar (PB3, PB5) at 3% and 5% (by mass) were applied. They were then mixed with the lateritic soil continuously until the color became uniform^65^. The mixed samples with 40% water content were incubated for half a month at 25 °C^66^. Soil without biochar amendment was set as control (CK). Soil pH, EC and CEC, plant available nutrients (e.g., K, P) were determined^61–64^. Soil EC was enhanced dramatically by 309.7–521.2% under unmodified biochar. Compared with unmodified biochar, P-modified biochar reduced the soil EC by 31.2–33.3%. Soil extractable N (from NO_3_^−^, NO_2_^−^, NH_4_^+^, etc.) was evaluated based on the methods proposed by Carter and Gregorich^67^, Jones and Willett^68^. Available anions (i.e., Cl^−^, NO_3_^−^, SO_4_^2−^, F^−^) in soil were measured using Ion Chromatograph proposed by Dick and Tabatabai^69^. The concentration of plant-available toxic metal Cd in soil was determined via the NH_4_NO_3_ extraction method^66^.

### Experimental set-up

The seed tubers of *P. heterophylla* in this study were supplied by Changhaojinhuang Traditional Chinese Medicine Co. LTD from Guizhou province, China. Seven seed tubers were planted at 60 mm depth with a horizontal spacing of 60 mm in each pot. The pots in this study were cylindrical with a diameter of 240 mm and a height of 160 mm^26^. The soil in each pot was compacted at relative compaction of 65% with the depth of 130 mm. For these five various treatments (CK, B3, PB3, B5, PB5), each soil condition had three replicated pots (21 seedlings in total). The adopted number of replicates followed the suggestions by Shetty and Prakash^70^, Silambarasan et al.^71^ and Zhou et al.^72^. A miniature-tip tensiometer and a moisture sensor were installed at 90 mm depth to monitor soil suction and volumetric water content (VWC) within the root depth zone respectively^73^.

All of the prepared pots were placed in a plant room with temperature of 28 ± 2 °C and humidity of 60 ± 5%. The cool white fluorescent lamps with light intensity of 200 μmol m^2^/s and wavelength of 400–700 nm were set in the top of pots as light source^74^. Lights for plants were turned on for 12 h and off for 12 h every day. During the 4-month plant growth period, the volumetric water contents of all vegetated soil pots were controlled (i.e., ~ 40%) by the irrigation every 4 days. No additional fertilizer (phosphorus, etc.) was provided during plant growth. No significant change of soil texture was observed during the experiment.

### Plant growth characteristics

During the plant growth period (4 months), leaf number, leaf area and shoot height were monitored every 2 weeks. Leaf area was obtained by ImageJ based on photographs while shoot height was measured by ruler. After 4 months, the plants were harvested and divided into different organs. The underground part of plants was gently washed by Milli-Q water to remove soil particles, which can minimize the potential damage. Root length was measured by ImageJ. Root length density was determined by dividing total length of roots over soil volume^75^.

### Yield and quality of root tuber

The measurement of tuber morphology including tuber length and maximum diameter of tuber was conducted by ImageJ^26^. The length of tuber was determined as the axial distance between the top of tuber and tuber tail. The maximum diameter was measured in the section with the largest lateral elongation. Based on Hong Kong Chinese Materia Medica standard^32^, the minimum diameter and length of tuber are larger than 2 mm and 10 mm, respectively. The wet and dry biomass of medicinal organ (root tuber) were measured before and after oven-drying at 60 °C for 24 h, respectively. Dry biomass of root tuber was used as the indicator of the yield^17^.

After oven-drying the root tubers and grinding them into powders, active ingredients were measured to evaluate the quality of *P. heterophylla*. Total polysaccharides in tuber of *P. heterophylla* were measured following anthrone sulfuric acid method proposed by HKCMMS (2020)^32^. Anthrone sulfuric acid solution was prepared by dissolving anthrone (0.1 g) in 80% sulfuric acid (100 mL). Firstly, the powdered samples of root tuber were mixed with distilled water for extraction of polysaccharides in water bath for 60 min. After centrifuging, the supernatant was collected. This extraction process was repeated for two more times. After combining the supernatants, ethanol was added to obtain the mixture at 4 °C for 12 h for precipitation of polysaccharides. The residue was dissolved in distilled water to obtain the test solution samples. Then, standard solution and test solution (2 mL) were mixed with anthrone sulfuric acid solution (6 mL) in a water bath (60 °C) for 15 min. After cooling in ice water bath for another 15 min, the mixture could be measured by UV/Visible spectrometer at 625 nm. Based on the standard of HKCMMS (2020)^32^, the concentrations of polysaccharides (calculated as anhydrous glucose) in dried plant samples should not be less than 6.0%.

Total saponins in tuber of *P. heterophylla* were measured by vanillin-ethanol method proposed by Zhang et al.^76^. The dried and powdered samples mixing with absolute ethanol were conducted by ultrasonic extraction. Ginsenoside Re (10 mg) was dissolved in methanol (100 mL) for the standard series. After drying the extraction (2 mL) and the standard solution with different volumes, 8% vanillin—ethanol solution (0.2 mL) and 60% sulfuric acid (5 mL) were added sequentially at 60 °C for 20 min. Finally, the concentrations of saponins in tested plant samples could be analysed after cooling by UV/Visible spectrometer at 560 nm.

### Total suction in root zone

Total suction in root zone soil was determined by the sum of matric suction and osmotic suction^77^. Matric suction was monitored by tensiometer during plant growth period and consequently its average value during the growth period could be obtained. Soil samples in the root zone at the initial and end of plant growth were collected. Osmotic suctions of these soil samples were estimated by the following Eq. ^62^:

$$\pi = 36EC_{meas} \omega_{ref} /\omega_{act}$$

where $\pi$ is the soil osmotic suction (kPa) at the average gravimetric water content ($\omega_{act}$, %) of the soil during plant growth period. $EC_{meas}$ is the measured electrical conductivity (dS/m) of the extraction from root zone soil at the reference gravimetric water content ($\omega_{ref}$, %) with the 1:5 soil/water mixture.

### Statistical analysis

The statistical package SPSS 20 (2011), the R software package (version 4.0.3) and Canoco 5.0 software were used for the statistical analysis in this study. Statistical differences between data from different soil conditions were assessed with one-way analysis of variance (ANOVA) using statistical package SPSS 20 (2011). Post-hoc Tukey’s honestly significant difference (HSD) was used in this analysis. Correlations were tested by Pearson’s correlation analysis. Results were considered to be statistically significant when *p* value was less than 0.05, corresponding to a 95% confidence interval. Different letters (e.g., a, b and c) were used to indicate statistical significance of difference (*p* < 0.05) among groups. Redundancy analysis (RDA) was implemented in Canoco 5.0.

## Supplementary Information

Supplementary Information.

## Data Availability

All data generated or analysed in this study are included in this published article and its supplementary information files.
