# Supplementary material for: Effects of phosphorus-modified biochar as a soil amendment on the growth and quality of Pseudostellaria heterophylla
Source: Sci Rep. 2022 May 4;12:7268. doi: 10.1038/s41598-022-11170-3 (PMC9066396; doi:10.1038/s41598-022-11170-3)
Supplement: Supplementary file 1 — Supplementary Information. [file 41598_2022_11170_MOESM1_ESM.pdf]

Effects of phosphorus-modified biochar as a soil amendment on the growth and quality of *Pseudostellaria heterophylla*

Charles Wang Wai NG [a], Yu Chen WANG [a, *], Jun Jun NI [a, *] and Pui San SO [a]

[a] Department of Civil and Environmental Engineering, the Hong Kong University of Science and Technology, Clear Water Bay, Hong Kong SAR, China

Charles Wang Wai NG

Department of Civil and Environmental Engineering, the Hong Kong University of Science and Technology, Clear Water Bay, Hong Kong SAR, China
(e-mail: charles.ng@ust.hk)

Yu Chen WANG

Department of Civil and Environmental Engineering, the Hong Kong University of Science and Technology, Clear Water Bay, Hong Kong SAR, China
*Corresponding author (e-mail: ycwangae@connect.ust.hk; telephone: 85256149560)

Jun Jun NI

Department of Civil and Environmental Engineering, the Hong Kong University of Science and Technology, Clear Water Bay, Hong Kong SAR, China
*Corresponding author (e-mail: cenijj@ust.hk)

Pui San SO

Department of Civil and Environmental Engineering, the Hong Kong University of Science and Technology, Clear Water Bay, Hong Kong SAR, China
(e-mail: psso@connect.ust.hk)

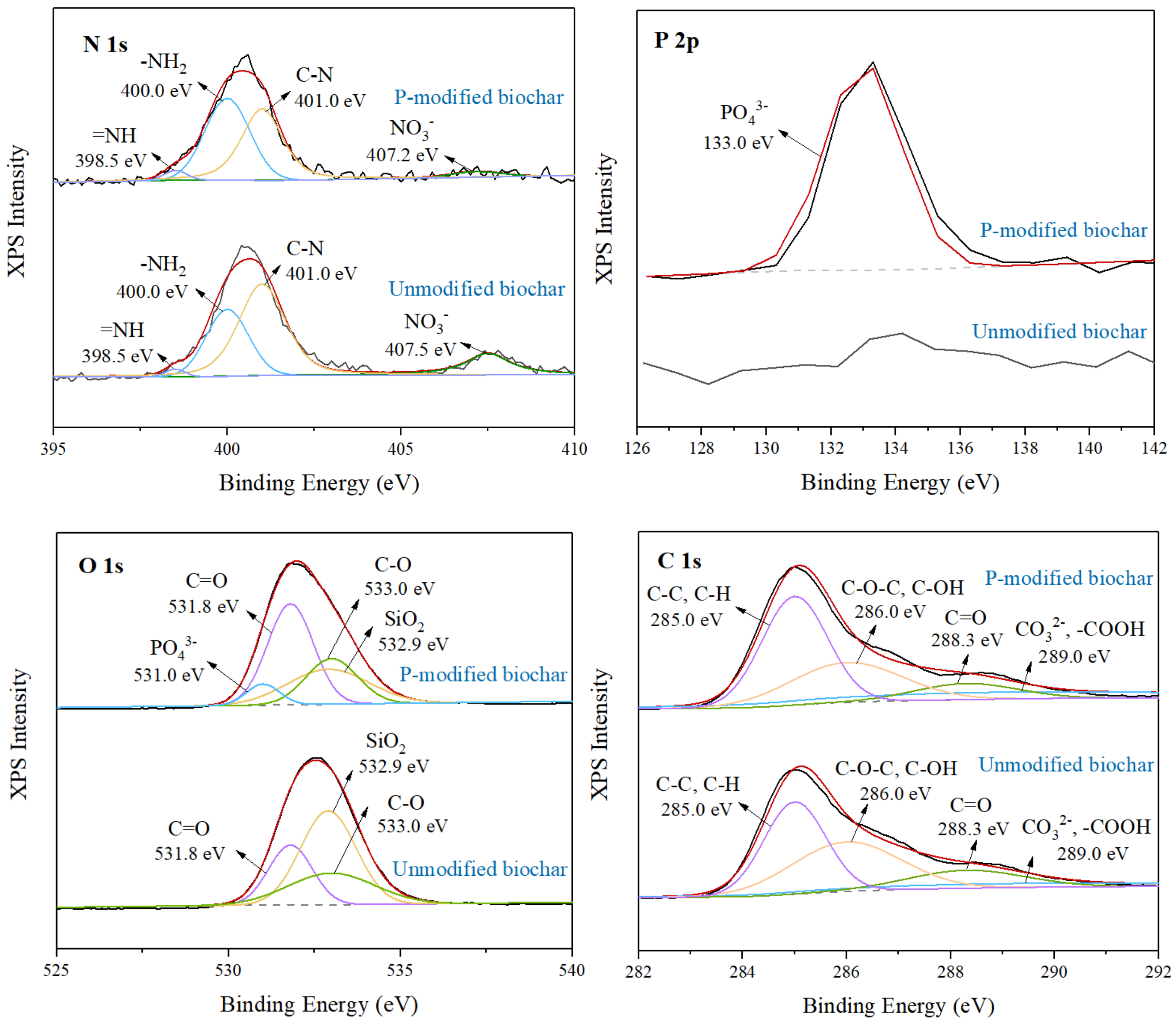

Figure S1 XPS deconvolutions of N1s, P2p, O1s, C1s for unmodified and P-modified biochar

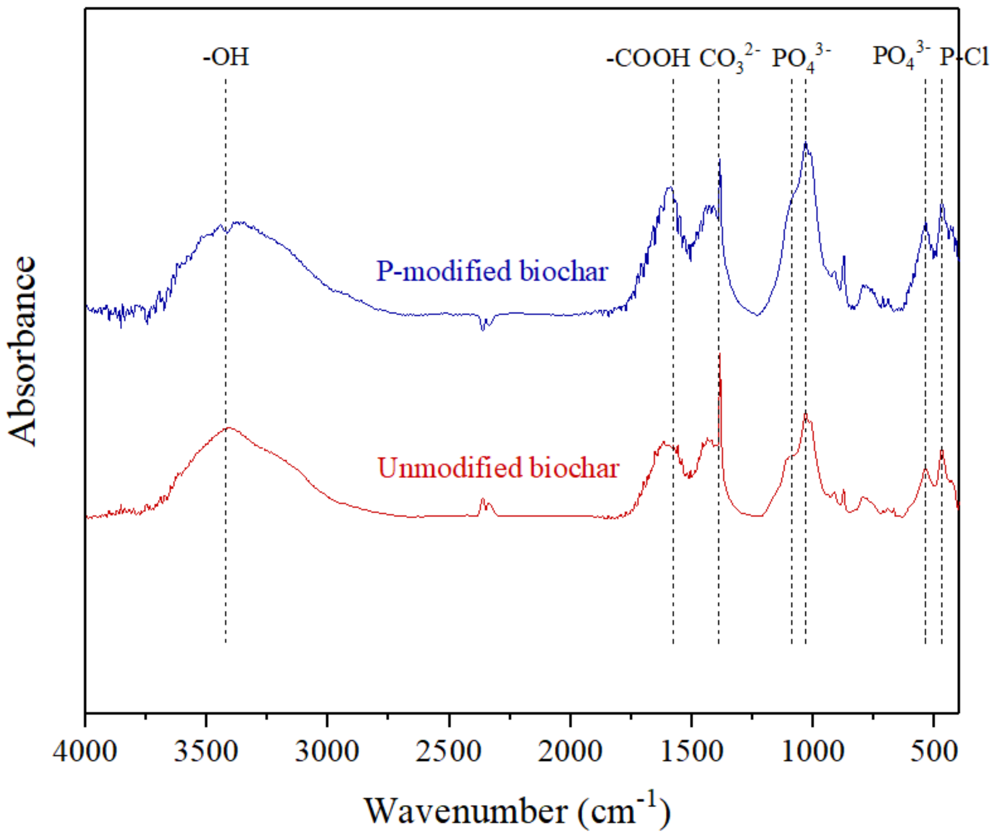

Figure S2 Fourier transform infrared spectra (FTIR) of unmodified and P-modified biochar

Table S1

Basic physicochemical properties of tested soil

Basic property	Value
pH	4.45±0.08
Electrical conductivity (μS/cm)	113.20±0.7
Cation exchange capacity (CEC) (meq/100g)	8.25±0.49
Total organic carbon (0.05%)	0.88
Plant available nutrients	
Potassium (K) (mg/kg)	90.07±7.86
Phosphorus (P) (mg/kg)	0.87±0.08
Magnesium (Mg) (mg/kg)	83.08±6.65
Calcium (Ca) (mg/kg)	402.10±53.19
Copper (Cu) (mg/kg)	2.77±0.43
Zinc (Zn) (mg/kg)	1.30±0.32

Table S2

Element composition of tested peanut shell biochar without and with modification

Element	Mass concentration (%)	
	Unmodified biochar	P-modified biochar
Mg	1.11	0.61
Na	0.83	0.55
Fe	2.07	1.49
O	33.63	33.03
N	3.22	2.36
Ca	5.16	8.23
C	36.88	37.15
K	1.93	3.09
Cl	3.08	1.07
Si	7.97	5.48
Al	4.10	2.81
P	-	4.13

Table S3

Initial electrical conductivity (EC) and cation exchange capacity (CEC) of soil under various treatments

Treatment	EC (μS/cm)	CEC (cmol(+)/kg)
CK	113	8.25
B3	463	10.51
PB3	309	11.26
B5	702	12.09
PB5	483	12.98
